# Development and validity of the Korea psychiatric triage algorithm

**DOI:** 10.1186/s12912-021-00738-5

**Published:** 2021-10-27

**Authors:** Jeongmin Ha, Kyeongmin Jang, Misuk An

**Affiliations:** 1 Department of Nursing, Chung-Ang University, 84 Heukseok-ro , Dongjak-gu, Seoul, Republic of Korea; 2 Department of Nursing, Bucheon University, 56, Sosa-ro, Bucheon-si, Gyeonggi-do Republic of Korea; 3 Heart Center, Chung-Ang University Hospital, 102, Heuseok-ro, Dongjak-gu, Seoul, 06973 Republic of Korea

**Keywords:** Psychiatric nursing, Triage, Algorithm

## Abstract

**Background:**

Psychiatric emergencies require timely intervention because of the risk of harm to individuals and society, including others. The aim of the present study was to test the content validity of a psychiatric triage algorithm developed for use in South Korea.

**Methods:**

The initial algorithm was developed through systematic literature review. Its validity was then verified by 10 experts. Based on results of expert validity, the algorithm was modified and the final algorithm was developed.

**Results:**

Its clinical validity was then verified by 37 emergency room nurses who had used triage. Four questions of expert validity results with a CVI of 0.8 or less were revised to reflect expert opinion. The usefulness, adequacy, and convenience of the final modified algorithm was 2.98 ~ 3.53.

**Conclusion:**

After sufficiently validated by follow-up studies, it is expected that the use of psychiatric classification algorithms in emergency room nurses will not only improve the quality of care, but also can improve patient outcomes and experience.

## Background

A psychiatric emergency is a situation in which emergency measures are required because people with various aggravated or acute illnesses are at risk of harming themselves or others [1]. Psychiatric emergencies, unlike physical emergencies, are notable because there is a risk of harm to individuals and the society, including others [2].

An increasing number of patients from all over the world visit emergency medical centers each year due to psychiatric emergencies [3]. In particular, Korea is ranked the 1st among Organization for Economic Cooperation and Development (OECD) countries with a suicide rate of 28.6%. Suicide death is one fatal consequence of psychiatric emergencies [4]. Therefore, the importance of intervention in psychiatric emergency patients is emerging [4].

In psychiatric emergencies, early detection of emergencies, rapid access to emergency care, and provision of specialized emergency mental health care should be provided organically [1]. Among these tasks, classifying the severity of psychiatric emergency patients by determining clinical characteristics quickly and accurately is the most important first step. This task is done by the emergency room (ER) nurse and is called triage. Patients who are misclassified as mild may deviate from medical attention and receive no immediate help, resulting in long stays in emergency medical centers [5]. which leads to overcrowding of the emergency medical center and burdens the patient with medical expenses [5, 6]. On the contrary, categorizing the severity of psychiatric emergency patients to be higher than it actually is can increase social costs to cover the health insurance [7], deteriorate the quality of nursing, and lower the satisfaction of both patients and employees [8].

Emergency medical center nurses often encounter patients with psychiatric emergencies [7], but they are trained on physical diseases as before [9]. Therefore, it is often more difficult to interpret psychiatric emergency patient’s response than when dealing with a patient who complains of physical problems [9]. Moreover, objective criteria and references to interpret psychiatric emergency patient’s response are limited [9].

In addition, nurses in emergency centers need to be accurate in their clinical situations, even in busy emergency situations. They must professionally deal with violent and aggressive behaviors of patients protesting over long waiting times for care [10]. Such an environment can be very stressful for professionals. The severity of psychiatric emergency patients may be misjudged by the prejudice of medical staff involved in the classification of subjects with psychiatric symptoms [10]. To more accurately classify psychiatric emergency patients at the clinical site, objective judgment criteria are needed [9].

Looking at Triage tools used in emergency rooms worldwide, Emergency Severity Index (US, 1999), Manchester Triage Scale (UK, 1997), Australian Triage Scale (AUSTRALIAN, 2001), Canadian Triage and Acuity Scale (CANADA, 1998), Cape Triage Score (South Africa, 2006). Each of these triage tools gives you maximum time to wait in the ER for each level. However, these triage tools are limited to emergency psychiatric disorders and are not developed, so there are limitations in their application to ER patients who are hospitalized for psychiatric problems.

Types of tools used to classify the severity of psychiatric emergency patients in emergency care settings vary by country. The Manchester Triage [11] and Canadian Triage and Acuity Scale (CTAS) [12] are typically used. Although these tools are suitable for physical emergencies that require preferential treatment for life-threatening symptoms, they are not entirely suitable for categorizing psychiatric emergencies that focus on social dysfunction [13]. For example, hostility, agitation, thought disturbance, positive symptoms of schizophrenia, suspiciousness and irritability, reduced social functioning, poor self-care in appearance, tone, and behavior can be immediately observed in psychiatric patients experiencing a psychiatric emergency [14]. However, these symptoms might not be real emergencies based on immediate life threats, although they can be exacerbated by self-harm or violence against others over time without proper treatment [15]. In addition, mental symptoms can be overlooked because they focus on solving physical problems when they are accompanied by mental and physical problems [13].

For this reason, Australia uses the Hobart Mental Health Triage Scale (MHTS) [16] and the South Eastern Sydney Area Health Service (SESAHS) [17], to classify patients with psychiatric emergencies into four levels and five levels, respectively. They categorize emergency situations to these levels and set the standard of maximum waiting time. South Eastern Sydney Area Health Service also categorizes symptoms to be observed or reported during classification. Although these tools could result in reduced waiting times in emergency centers, they did not include physical symptoms, making it difficult to judge situations involving both mental and physical symptoms [18].

The Crisis and Triage Rating Scale (CTRS) [19] was developed in the United Kingdom. It was first introduced in Korea in 2005 for crisis management for mentally ill patients in the community. It is currently being used in mental health promotion centers [20]. This measure has three categories: risk, support system, and cooperative capacity. It showed a high predictive validity for psychiatric emergency patients [19]. With this measure, subjects who receive less than 9 points are suggested to be hospitalized [19]. However, CTRS is not really a risk-based classification, but rather an inpatient and discharge of the patient. It does not include physical conditions that accompany mental emergencies [21]. In addition, there is a limit in measuring the severity if the patient is not cooperative because knowledge of the patient’s social and family support system is required [18].

Color-Risk Psychiatric Triage [18] was an algorithm developed in Mexico in 2016. Physical assessments were performed first, followed by psychological evaluation. Actual or potential risks were considered to be the main cause of psychiatric emergency classification. Color-Risk Psychiatric Triage’s classification criteria are accurate. It is easy to check the severity of the classification using color codes that indicate priority [18]. However, as a severity classification tool for doctors, an appropriate prescription is provided for each severity, which is somewhat different from the nurse’s triage work. In addition, it is complicated to use for triage.

In the emergency room, the time it takes for a patient to see a doctor depends on the outcome of the triage performed by the nurse. Triage is administered prior to the face of the doctor and is not intended for prescription. That is, the prescription obtained through this does not meet the purpose of the triage administered by the nurse, so it is inappropriate to utilize Color-Risk Psychiatric Triage. This requires the development and evaluation of severity classification criteria for use quickly and easily by nurses working in triage during psychiatric emergencies.

Thus, the purpose of this study was to develop a triage algorithm to determine the severity of patients who visited the ER due to psychiatric emergencies.

## Methods

### Design

To develop Triage algorithm for determining the severity of patients who visited the emergency room due to psychiatric emergencies, the literature was reviewed and experts’ CVIs were checked, and the opinions of clinical nurses were confirmed on the usefulness, adequacy, and convenience of the developed algorithm.

### Ethical consideration

This study was conducted after receiving Institutional Review Board (IRB) approval from B** Medical Center (IRB No. 20190729/30–2019-74/083).

### Research process

#### Initial algorithm development

This study systematically reviewed the literature on psychiatric emergency patient classification tools to develop algorithms. PubMed, Embase, Cochrane Central Register of Controlled Trials (CENTRAL), CINAHL, Web of Science, SCOPUS, ProQuest Dissertations & Theses Global (PQDT Global), KoreaMed, KMBASE, KISS, DBPia, and NDSL were searched. Google scholar articles in the last 10 years was searched using key terms of ‘Mental health illness’, ‘psychiatric emergency’, ‘triage’, ‘decision aid’, ‘assessment’, ‘Validity’, ‘reliability’, ‘algorithm’ by combining AND and OR. Based on quality evaluation of selected documents, three researchers revised items and paths of the algorithm suitable for Korean situation through three meetings.

#### Validation and correction of content validity for initial algorithm

For content validity, eight to twelve expert panels are recommended to be established [22]. Therefore, in this study, a total of 10 expert panels were formed, including two specialists in emergency medicine, one psychiatrist, two mental health specialist with over 10 years of experience in a psychiatric ward as nurses, two nurses with more than 10 years of experience in emergency medical center, one mental health specialist with over 10 years of experience in emergency department as a social worker. To collect data, experts were sent an e-mail with a description of the study and a consent form. They were asked to reply if they would like to participate in the study within 2 weeks. In the case of a reply, the researcher set up an appointment with an expert, met with the expert, and collected the consent form and questionnaire. Initial algorithms were presented to 10 experts for content validity. After that, we checked the validity of the initial algorithm through 16 questions. Based on prior literature [23], for each question, participants were asked to rate 1 to 4 points, ranging from ‘not very appropriate’ to ‘very appropriate’. For additional comments, a blank space was provided for each item in the questionnaire.

#### The usefulness, adequacy, and convenience of the modified algorithm

In considering test use, the practitioners must also consider the opinions of clinical nurses on the usefulness of the modified algorithm [24]. These test properties refer to the accuracy with which a test identifies a patient’s clinical status. Confirm the usefulness, adequacy, and convenience of the developed algorithm, 25 patients were classified as severe based on previous studies [25]. A total of 37 data were collected considering the possibility of withdrawal from the study. To verify the usefulness, adequacy, and convenience of the modified algorithm, a total of 37 nurses (14 nurses and 23 nurses who performed triage in the emergency department of C University Hospital and B University Hospital in Seoul, respectively) participated in this study. Recruitment notices, a description of the study, and a consent form were kept in the break room to voluntarily complete the questionnaire and put it in the collection box. Through this method, nurses participating in the study were informed of this study and voluntarily agreed to participate in the study. Evaluation of the usefulness, adequacy, and convenience of the algorithm was carried out using a tool developed by Paul [1999]. For each question, participants were asked to rate 1 to 4 points, ranging from ‘not so’ to ‘Really so’. For additional opinions, blank space was left for each question in the questionnaire. The higher the score, the higher the practical suitability of the algorithm. Nurses who participated in this study evaluated the clinical validity of the modified algorithm based on their experience of classifying patients who visited the ER due to psychiatric problems. All 30 questionnaires were used for analysis without withdrawal from the study or unfaithful responses.

#### Final algorithm confirmation

**Fig. 1:**
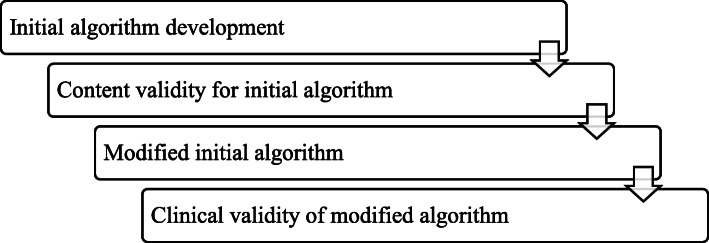
Research process

The final algorithm was confirmed by verifying that there was no difficulty or problem for nurses to apply the algorithm to patients through practical suitability verification of the modified algorithm. Figure 1 shows the research process.

### Data analysis

To confirm the content validity of the initial algorithm, CVI was calculated for each item. Based on Lynn [1986], the content validity index (CVI) was calculated for 16 items. Items with CVI of 0.8 or higher were selected. Items with CVI below 0.8 were supplemented by reflecting the revised opinion provided by experts.

To confirm the usefulness, adequacy, and convenience of the modified algorithm, average frequency and standard deviation of the algorithm’s frequency of use, usefulness, adequacy, and convenience were calculated.

## Results

### Initial algorithm development

**Fig. 2:**
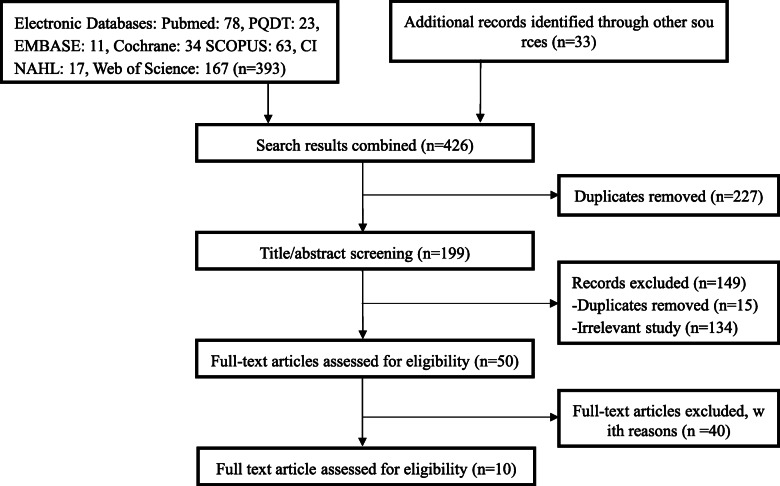
PRISMA flow chart of study selection

A total of ten documents were searched (Fig. 2).

### Content validity of initial algorithm

The expert group to verify the validity of the initial algorithms included two professors of mental health medicine, two professors of emergency medicine, one head nurse, one nurse who had worked in a psychiatric ward for more than 10 years, two who had worked in an ER for more than 10 years, one suicide case manager, and one social worker. Two of them had bachelor’s degrees. Four of them had master’s degrees. Four of them had doctoral degrees. The average working experience was 16.3 ± 8.5 years.

Content validity test of the expert group on the initial algorithm revealed 11 items (2, 2–1, 2–2, 3, 4, 4–1, 4–3, 5, 5–1, 6, 6–2) with CVI values above 0.9, one item (3–1) with CVI value above 0.8, and four items (1, 4–2, 5–2, 6–1) with CVI value below 0.8 (Table 1).

**Table 1:** Expert Content Validation Results for the Initial Algorithm (*N* = 10)

No.	Item	CVI
1.	Is it reasonable to distinguish between mental and physical problems with the patient’s chief complaint?	.6
2.	Is it appropriate to confirm that the physical problem of the patient who came to the emergency room is the result of suicide attempt?	.9
2–1.	Is it reasonable to classify an emergency if a physical problem is the result of a suicide attempt?	1.0
2–2.	Is it reasonable to use KTAS if physical problems are not the result of suicide attempts?	1.0
3.	If aggressive behavior is observed, is it reasonable to classify as urgent?	1.0
3–1.	Is it reasonable to have a psychiatrist’s face-to-face medical treatment immediately in an urgent?	.8
4.	Is it reasonable to classify as an emergency if you have severe agitation / suicide attempts?	1.0
4–1.	The criteria for severe agitation are based on the Excited Component of the Positive and Negative Syndrome Scale. Is the classifier valid?	1.0
4–2.	Is it appropriate to have a psychiatrist visit in 15 to 30 min in an emergency?	.7
4–3.	Is it appropriate to report to an emergency medical specialist if an emergency is found that causes physical damage due to suicide attempts?	.9
5.	Is it reasonable to classify as a para-emergency if you show mild agitation?	1.0
5–1.	The criteria for mild agitation are based on the Excited Component of the Positive and Negative Syndrome Scale. Is the classifier valid?	1.0
5–2.	Is it reasonable to have a psychiatrist visit within 30 to 60 min if you are para-emergency?	.7
6.	Is the non-emergency classifier valid?	1.0
6–1.	If it is non-emergency, is it reasonable to have a psychiatrist visit within 60 to 120 min?	.6
6–2.	If it is non-emergency, is it reasonable to make a discharge or outpatient appointment if the patient does not complain of a subjective psychiatric emergency?	1.0

*KTAS* Korean triage and acute scale

### Expert opinion on initial algorithms

For item 1 (Is it appropriate to distinguish between mental and physical problems by the patient’s address?), there was an opinion that it could be difficult. In the case of suspicion of appealing psychiatric problems as a result of physical problems, there was an opinion that it was necessary to consider physical causes rather than approach psychiatric problems. There were also opinions that it would be more appropriate to classify based on address and past history and that there was a need to prioritize patients who had a complex problem.

For item 4–2 (Would it be appropriate to have a psychiatrist visit within 15 to 30 min in case of an emergency?), there was an opinion that it would be realistic to treat a resident within 30 ~ 60 min of a psychiatric emergency because it would be practically difficult if the resident did not reside in the emergency room.

For question 5–2 (Is it appropriate to have a psychiatrist visit within 30 to 60 min in case of para-emergency?), in the case of para-emergency, there was an opinion that face-to-face medical treatment of emergency medical specialists or residents who resided in the ER would be more realistic than psychiatrist within 30 to 60 min.

**Fig. 3:**
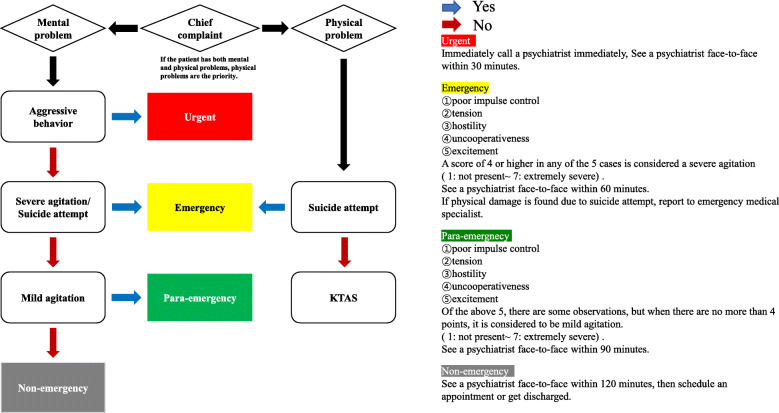
Modified algorithm

For question 6–1 (Is it appropriate to have a psychiatrist visit within 60 to 120 min for non-emergencies?), in the case of non-emergency, there was an opinion that it would be appropriate to visit the resident outpatient after the resident or the emergency clinic within 2 h rather than the psychiatrist. Based on results of content validity analysis of these expert groups, the modified algorithm was derived by modifying and supplementing the initial algorithm (Fig. 3).

### The usefulness, adequacy, and convenience of the modified algorithm

General characteristics of the triage nurse group for clinical validation of the modified algorithm are as follows (Table 2). There were 7 (18.9%) males and 30 (81.1%) females. Their mean age was 33.41 ± 5.17 years. Regarding the educational level, there were 9 (24.3%) college graduates, 24 (65.9%) with bachelor’s degrees, and 4 (10.8%) with master’s degrees or higher. Their average clinical experience was 89.21 ± 45.32 months. Total ER experience was 81.11 ± 47.21 months.

**Table 2:** General Characteristics of the Triage Nurse Group (*N* = 37)

Variable	N (%)	Mean (SD)
Sex		
Male	7 (18.9)	
Female	30 (81.1)	
Age		33.41 (5.17)
Education level		
College graduation	9 (24.3)	
Bachelor’s degrees	24 (65.9)	
Master’s degrees or more	4 (10.8)	
Total clinical experience (Month)		89.21 (45.32)
Total emergency room experience (Month)		81.11 (47.21)

*SD* Standard deviation

The average rating of items to validate clinical validity was 2.98 ± 0.67 ~ 3.53 ± 0.65. Items that the algorithm helped in nursing activities and the importance of the algorithm had the highest ratings (3.47 ± 0.57 and 3.53 ± 0.65, respectively). On the other hand, the degree to which the algorithm helped in cost-effectiveness was relatively low (average 2.98 ± 0.67) (Table 3).

**Table 3:** Algorithm Usefulness, Adequacy, and Convenience (*N* = 37)

Items	Mean (SD)
1. Is the algorithm appropriate for psychiatric crisis screening?	3.32 (.57)
1–1) Was the term easy to understand?	3.42 (.69)
1–2) Was it easy to use?	3.37 (.61)
2. Did the algorithm help with nursing activities?	3.47 (.57)
3. Did the algorithm save time in nursing activities?	3.22 (.44)
4. Was the algorithm cost effective for nursing activities?	2.98 (.67)
5. Are the algorithms scientific and systematic?	3.33 (.73)
6. How important do you think the algorithm is?	3.53 (.65)

*SD* Standard deviation

## Discussion

The purpose of this study was to develop an algorithm for evaluating the severity of patients who experienced psychiatric crisis in Korean emergency room and to find out whether it could be applicable to emergency room nurses. For algorithm development, the initial algorithm was revised after verifying the expert validity by considering previous studies. The usefulness, adequacy, and convenience were then tested by educating emergency room nurses to use the modified algorithm and evaluating it based on previous experience.

For Item 1 (Physical problems are prioritized when both mental and physical problems are appealed.), the expert content validity index was less than 0.8. Thus, the severity classification of psychiatric emergency patients with physical problems can be achieved more consistently with this tool than with the Crisis and Triage Rating Scale [19] which does not consider physical problems. This allows us to prioritize patients with complex problems. In addition, for items 4–2, 5–2, and 6–1, considering the situation in Korea where psychiatrist does not reside in the emergency room, the psychiatrist, not the resident, performs face-to-face treatment. The time required for face-to-face medical treatment is too short. This point was considered to be lacking the reality. Therefore, the criteria were changed to ‘face-to-face treatment by resident within 60 minutes in case emergency’ for 4–2, ‘face-to-face treatment by resident within 90 minutes in case of para-emergency’ for item 5–2, and ‘face-to-face treatment by resident within 120 minutes in case non-emergency’ for item 6–1. Through this, the effectiveness of the algorithm was improved so that it could be applicable to the clinical field.

After clinical practitioners received training on modified and complementary algorithms, the practical suitability was verified based on the experience of classifying patients who experienced psychiatric crisis. As a result, the item that the algorithm helped nursing activities and the importance of the algorithm scored 3.47 points out of 4 points. Algorithms can often be used for nurses’ clinical judgment or nursing decision making. Algorithms are visually organized, easy to focus on, easy to understand and help nurses acquire knowledge for problem-solving [26]. While the patient’s severity classification varied greatly according to nurses’ individual competencies, the modified algorithm seems to be helpful in practice by providing a consistent and unified nursing provision and suggesting theoretical basis and criteria that are easy to perform.

In addition, the item ‘was the term easy to understand?’ scored 3.37 which was a relatively high score. Korean Triage and Acuity Scale previously used in Korea had a different classification according to working experience of ER nurses due to an ambiguous standard setting [27]. In particular, the training for nurses in the ER is mainly about physical symptoms [9], making it difficult to determine the severity of psychiatric symptoms by the ambiguous standard of Korean Triage and Acuity Scale.

Therefore, we tried to improve problems of the existing Korean Triage and Acuity Scale in the development of the algorithm of this study. First, to clarify the criterion for the concept of ‘agitation’, which was a highly abstract concept among algorithms, the criteria for ‘agitation’ were presented based on the Positive and Negative Syndrome Scale (PANSS-EC) [28]. In addition, this study was written in one chapter to simplify the explanation of the algorithm path and the reference that could be used when using the algorithm. By providing detailed criteria on one side of the algorithm, it is possible to increase the consistency of performance based on the rationale without disturbing the clarity of the algorithm path.

On the other hand, the questionnaire asking whether the algorithm had cost-saving effect scored the lowest at 2.9 points. Items asking whether the algorithm reduced time in nursing activities scored 3.13 points. Items asking whether it was easy to use scored 3.13 points, which was relatively low. Stages for dividing psychiatric emergencies varied among triage tools. Although as many as seven stages have been distinguished [14], there are generally four to five stages. The Color-Risk Psychiatric Triage used in the ER to classify the severity of patients experiencing psychiatric crises was developed to address only psychiatric crises. Therefore, it is an algorithm that understands and reflects psychiatric crisis well. The Color-Risk Psychiatric Triage divides the psychiatric crisis into five stages [18].

For those with phases 2 and 3 showing no difference in mediation, the psychiatrist should treat them face to face within 15 to 30 min [18]. Triage nurses must quickly and accurately classify patients with limited information in complex, dynamic decision-making processes in a fraction of the time. To simplify the decision-making stage according to the role of the triage nurse, the algorithm consisted of 4 stages of psychiatric crisis by integrating stages 2 and 3.

Despite these considerations, the cost-effectiveness could have been underestimated because the algorithm was not yet familiar to nurses at the time of validating the clinical validity, making it more time-consuming and difficult for nurses to use it for making decisions. In fact, two nurses wrote their comments “It’s easy to use, but you have to watch it every time. I think it will improve if I learn it through repeated use”. Also, because the algorithm was not computerized, nurses might have felt inefficient. In fact, there was an opinion that ‘computerization is required for faster access for use in the emergency room’. Therefore, it is necessary to reevaluate ER nurses who are accustomed to using the algorithm after computerizing the algorithm developed in this study. There is also a need to look at the long-term cost-effective aspect. Prior studies have shown that determining the appropriate severity of ER patients helps reduce the length of ER stay and medical costs for ER patients [5].

This study has several limitations. First, we calculated the CVI value based on the opinions of experts and modified the algorithm to reflect the experts’ comments, but did not go through the process of reviewing it again. And second, we have found it difficult to recruit and include more participants in the pilot phase of this validity study. Therefore, there is a descriptive analysis that hampers a deeper interpretation of the algorithm’s performance. If the sample size has improved, that would strengthen the study. Additionally, due to the scarcity of available data, the statistics (CVI analysis) just could describe a facet of the proposed question. It is recommended a systematic item analysis, e.g., the probability of the endorsement rate by the attending nurse in ER and further comparison with a robust assessment will ensure its “criterion validity”. And third, the cost-effectiveness of the algorithm developed in this study could not be confirmed and the feasibility analysis was not performed on patients who visited the emergency room. Thus, it is early and hasty to conclude that this tool is useful for detecting severity of psychiatric conditions in ER. More data and analyses should be included to demonstrate the study’s goal. It is necessary to conduct a study that compares outcome variables such as the length of the ER stay before and after applying the algorithm developed in this study, or the degree of agreement between results of the nurse’s triage and hospitalization.

## Conclusions

In this study, we developed an algorithm based on literature review and verified its validity using expert content validity. Through the use of the algorithm developed in this study, it is expected that the severity of psychiatric crisis patients who visit the ER can be systematically and consistently classified. It will help reduce the length of stay of patients. It can also help ensure patient safety and save unnecessary medical and social expenses. Before that, we suggest a follow-up study to confirm sufficient validity.

## Data Availability

The datasets used and/or analysed during the current study are available from the corresponding author upon reasonable request.

## Abbreviations

ER: Emergency room
OECD: Organization for economic cooperation and development
CTAS: Canadian triage and acuity scale
MHTS: Hobart mental health triage scale
SESAHS: South eastern Sydney area health service
CRTS: The crisis and triage rating scale
IRB: Institutional review board
CVI: Content validity index

## References

[1] Shin SD, Hahm BJ, Song KJ, Hwang SS, An KO, Park JO, et al. In: Model development of psychiatric emergency medical service system. Seoul: National Center for Mental Health; 2010. p. 47-48.

[2] Sudarsanan S, Chaudhury S, Pawar A, Salujha S, Srivastava K (2004). Psychiatric emergencies. Med J Armed Forces India.

[3] Weiss AJ, Barrett ML, Heslin KC, Stocks C (2006). Trends in Emergency Department visits involving mental and substance use disorders, 2006–2013: statistical brief# 216. Healthcare Cost and Utilization Project (HCUP) Statistical Briefs [Internet].

[4] Hong J, Lee D, Ham B, Lee S, Sung S, Yoon T (2017). The survey of mental disorders in Korea 2016. Ministry of Health and Welfare, Seoul (Korea).

[5] Cremonesi P, di Bella E, Montefiori M, Persico L (2015). The robustness and effectiveness of the triage system at times of overcrowding and the extra costs due to inappropriate use of emergency departments. Appl Health Econ Health Policy.

[6] Yang KK, Lam SSW, Low JM, Ong MEH (2016). Managing emergency department crowding through improved triaging and resource allocation. Operations Res Health Care.

[7] Woo JH, Grinspan Z, Shapiro J, Rhee SY (2016). Frequent users of hospital emergency departments in Korea characterized by claims data from the National Health Insurance: a cross sectional study. PLoS One.

[8] Varjoshani NJ, Hosseini MA, Khankeh HR, Ahmadi F (2015). Tumultuous atmosphere (physical, mental), the main barrier to emergency department inter-professional communication. Global J Health Sci.

[9] Plant LD, White JH (2013). Emergency room psychiatric services: a qualitative study of nurses’ experiences. Issues Mental Health Nurs.

[10] Lacy J (2011). Improving the assessment and triage of patients with mental illness attending the emergency department [masters dissertation].

[11] Wulp IV, Baar ME, Schrijvers AJP (2008). A. Schrijvers. Reliability and validity of the Manchester triage system in a general emergency department patient population in the Netherlands: results of a simulation study. Emerg Med J.

[12] Beveridge R, Ducharme J, Janes L, Beaulieu S, Walter S (1999). Reliability of the Canadian emergency department triage and acuity scale: interrater agreement. Ann Emerg Med.

[13] Downey LVA, Zun LS, Burke T (2015). Comparison of Canadian triage acuity scale to Australian emergency mental health scale triage system for psychiatric patients. Int Emerg Nurs.

[14] Sands N, Elsom S, Colgate R, Haylor H, Prematunga R (2016). Development and interrater reliability of the UK mental health triage scale. Int J Ment Health Nurs.

[15] Zeller SL (2010). Treatment of psychiatric patients in emergency settings. Prim Psychiatry.

[16] Smart D, Pollard C, Walpole B (1999). Mental health triage in emergency medicine. Aust N Z J Psychiatry.

[17] Broadbent M, Moxham L, Dwyer T (2007). The development and use of mental health triage scales in Australia. Int J Ment Health Nurs.

[18] Molina-López A, Cruz-Islas JB, Palma-Cortés M, Guizar-Sánchez DP, Garfias-Rau CY, Ontiveros-Uribe MP, Fresán-Orellana A (2016). Validity and reliability of a novel color-risk psychiatric triage in a psychiatric emergency department. BMC Psychiatry.

[19] Bengelsdorf H, Levy LE, Emerson RL, Barile FA (1984). A crisis triage rating scale: brief dispositional assessment of patients at risk for hospitalization. J Nerv Ment Dis.

[20] Ministry of Health and Welfare. (2019). Mental Emergency Manual. Retrieved from http://www.mohw.go.kr/react/al/sal0301vw.jsp? PAR_MENU_ID=04&ME NU_ID=0404&CONT_SEQ=349148.

[21] Turner P, Turner T (1991). Validation of the crisis triage rating scale for psychiatric emergencies. Can J Psychiatr.

[22] Polit DF, Beck C (2010). Essentials of nursing research: Philadelphia: Wolters Kluwer health/Lippincott Williams & Wilkins.

[23] Holtzman NA, Watson MS (1999). Promoting safe and effective genetic testing in the United States. Final report of the task force on genetic testing. J Child Fam Nurs.

[24] Kim JM, Park JS (2010). Development of an algorithm for the prevention and management of pressure ulcers. Korean J Adult Nurs.

[25] Song Y-Y, Ying L (2015). Decision tree methods: applications for classification and prediction. Shanghai Arch Psychiatry.

[26] Moon SH, Park YH (2018). Development of a triage competency scale for emergency nurses. J Korean Acad Nurs.

[27] Montoya A, Valladares A, Lizán L, San L, Escobar R, Paz S (2011). Validation of the excited component of the positive and negative syndrome scale (PANSS-EC) in a naturalistic sample of 278 patients with acute psychosis and agitation in a psychiatric emergency room. Health Qual Life Outcomes.

